# Preliminary Evidence Linking Maternal Sleep-Disordered Breathing During Pregnancy to Early Childhood Development: A 3-Year Pilot Cohort Study in Japan

**DOI:** 10.3390/children12121610

**Published:** 2025-11-26

**Authors:** Yu Takenouchi, Jun Hosomichi, Takumi Suzuki, Mayu Niisaka, Naoyuki Miyasaka, Chikako Morioka, Manabu Sugie, Mari Hayata, Jun Aida, Meiyo Tamaoka, Yasunari Miyazaki, Takashi Ono

**Affiliations:** 1 Department of Orthodontic Science, Graduate School of Medical and Dental Sciences, Institute of Science Tokyo, Tokyo 113-8510, Japan; 2 Department of Perinatal and Women’s Medicine, Graduate School of Medical and Dental Sciences, Institute of Science Tokyo, Tokyo 113-8510, Japan; 3 Department of Pediatrics and Developmental Biology, Graduate School of Medical and Dental Sciences, Institute of Science Tokyo, Tokyo 113-8510, Japan; 4 Department of Dental Public Health, Graduate School of Medical and Dental Sciences, Institute of Science Tokyo, Tokyo 113-8510, Japan; 5 Department of Respiratory Medicine, Respiratory Center, Toranomon Hospital, Tokyo 105-8470, Japan; 6 Department of Respiratory Medicine, Institute of Science Tokyo, Tokyo 113-8510, Japan

**Keywords:** maternal obstructive sleep apnea, pregnancy, offspring development, growth, neurodevelopment

## Abstract

**Highlights:**

**What are the main findings?**

- Obstructive sleep apnea (OSA) during pregnancy may be associated with lower cognitive and language development in 3-year-old children.
- No clear associations were found with children’s physical growth or occlusal status.
- Early screening for maternal sleep-disordered breathing using home sleep tests may help identify high-risk pregnancies.

**What are the implications of the main findings?**

- Raising awareness on maternal sleep health may help prevent developmental differences during early childhood.
- Feasible prenatal screening tools, such as home sleep apnea testing, could enable early identification and management of maternal OSA.
- Integrating sleep health assessments into routine prenatal care may improve long-term outcomes for both mothers and children.

**Abstract:**

Background/Objectives: Obstructive sleep apnea (OSA) during pregnancy may reduce maternal oxygenation, cause sleep fragmentation, and influence offspring development. This pilot study explored potential associations between OSA during pregnancy and child outcomes at age 3. Methods: Pregnant women aged 23–48 years who underwent home sleep apnea testing (HSAT) at 28–32 weeks of gestation between June 2021 and July 2025 were enrolled. OSA was defined as an apnea–hypopnea index (AHI) ≥ 5. Mothers and children were prospectively followed until the child reached 3 years of age. Children’s developmental levels (motor, cognitive/adaptive, language/social, and total) were evaluated using the New K-Type Developmental Test. Anthropometric measures (height, weight, and head circumference) and dental occlusion were also assessed. Correlations between the maternal AHI and developmental indices were examined. Results: Thirty-four women, including six with OSA, completed the follow-up assessment. No significant differences were observed in children’s physical growth or occlusion between the OSA and non-OSA groups. The maternal AHI showed a negative tendency with the total developmental index and the cognitive/adaptive and language/social domains. One participant with severe OSA (AHI = 69.3) showed markedly lower developmental scores, suggesting a possible dose-dependent trend rather than a definitive threshold. Given the small number of OSA cases and the influence of a single severe case, these findings should be interpreted cautiously as preliminary and descriptive. Conclusions: OSA during pregnancy may be associated with differences in early childhood development. The findings highlight the importance of maternal sleep health awareness and feasible screening approaches, such as HSAT, during pregnancy.

## 1. Introduction

Maternal sleep-disordered breathing (SDB), including obstructive sleep apnea (OSA), has recently gained attention within the Developmental Origins of Health and Disease (DOHaD) framework, which emphasizes that maternal health and intrauterine exposures may influence the health and neurodevelopment of offspring across their lifespan [1,2,3,4]. OSA involves recurrent upper airway obstruction during sleep, leading to intermittent hypoxemia and sleep fragmentation. Physiological changes during pregnancy—such as increased ventilatory drive from progesterone, gestational weight gain, and upper airway edema—can predispose women to the onset or worsening of OSA [5,6]. Thus, maternal OSA offers a biologically plausible context to explore how maternal hypoxic and inflammatory stressors may contribute to fetal brain development.

Epidemiological data suggest that up to 20% of pregnant women experience some degree of OSA, with the prevalence increasing in the third trimester [7,8]. However, mild OSA predominates, and moderate-to-severe cases remain relatively uncommon (<5%) [9,10,11]. While prior research has primarily focused on perinatal complications [5,12,13], the potential long-term developmental implications of maternal OSA, particularly regarding domain-specific neurocognitive outcomes in children, have not been fully characterized.

Mechanistically, maternal OSA can cause intermittent hypoxemia, oxidative stress, and vascular remodeling, which may impair placental oxygen and nutrient transport. These alterations can trigger systemic and placental inflammation involving cytokines such as interleukin-6, tumor necrosis factor-α, and C-reactive protein, all of which may cross the placental barrier and influence fetal neural development [5,14,15,16]. Chronic maternal hypoxia and inflammation may also induce epigenetic modifications—altered DNA methylation and microRNA expression—that have been linked to long-term neurodevelopmental differences in offspring [17,18,19,20]. Collectively, these biological pathways suggest that maternal OSA could represent a potentially modifiable prenatal risk factor for child neurodevelopmental variation [21,22,23].

Few longitudinal studies have reported associations between maternal OSA and adverse child outcomes, such as low birth weight, preterm birth, and behavioral or attention-related difficulties later in life [24,25,26,27]. However, most of these studies were constrained by small sample sizes, a lack of comprehensive adjustment for confounders, and reliance on parent-reported questionnaires, which are susceptible to recall bias [5,10]. Objective, examiner-administered developmental assessments are needed to clarify how maternal OSA may relate to multidimensional aspects of early child development, including motor, cognitive, language, and social domains [28,29,30,31].

Furthermore, most existing evidence originates from Western populations [32,33]. Cultural and healthcare differences, such as maternal age, perinatal care systems, and early-childhood health checkups, may limit generalizability to Asian contexts. Given its higher maternal age at delivery [34], increasing rates of obesity [35], and standardized pediatric health examinations that enable objective developmental evaluations [36], Japan represents a valuable setting for such investigations.

From both clinical and public health perspectives, understanding how maternal OSA may influence child development is increasingly important. Early identification and management of maternal SDB could offer a feasible opportunity to promote maternal and child well-being [37,38]. Therefore, this prospective pilot cohort study aimed to explore potential associations between maternal OSA during pregnancy and developmental outcomes of children at 3 years of age in a Japanese population using an examiner-administered, domain-specific developmental test to provide preliminary insights into the intergenerational effects of maternal sleep health.

## 2. Materials and Methods

### 2.1. Study Design and Participants

This exploratory prospective cohort study enrolled pregnant women who delivered at the Department of Obstetrics and Gynecology, Institute of Science Tokyo Hospital (Japan), between 15 June 2021, and 18 July 2025. Maternal sleep and breathing were assessed using home sleep apnea testing (HSAT) at 28–32 weeks of gestation [39]. Mothers and their children were followed until the child reached 3 years of age, at which point developmental assessments were conducted. All children who completed the follow-up assessment were born between 27 July 2021, and 15 July 2022.

All participants provided written informed consent after receiving detailed explanations of the study purpose and procedures. Exclusion criteria included (i) a lack of consent, (ii) inability to obtain valid apnea–hypopnea index (AHI) data [40], or (iii) inability to complete the 3-year developmental follow-up.

This study was approved by the Institutional Review Board of the Institute of Science Tokyo Hospital (approval no. G2020-037, 23 March 2021) and was conducted in accordance with the Declaration of Helsinki and national ethical guidelines.

### 2.2. Maternal Characteristics and Questionnaire

Maternal demographic and clinical information—including maternal age at delivery, parity, smoking history (current, past, or none), pre-pregnancy body mass index (BMI), systemic comorbidities (e.g., hypertension, diabetes, and bronchial asthma), and perinatal complications—was obtained using a structured, self-administered questionnaire. These data were cross-checked against medical records for accuracy.

### 2.3. Assessment of Maternal OSA

HSAT was conducted between 28 and 32 weeks of gestation using a validated portable device (WatchPAT; Philips, Murrysville, PA, USA). The WatchPAT device has demonstrated acceptable agreement with in-laboratory polysomnography (PSG) in pregnant populations for detecting obstructive events and estimating the AHI [39,41]. However, previous studies indicated that HSAT may slightly underestimate mild OSA compared with PSG, particularly in positional or REM-related cases [39,42].

PSG was not employed in this study because overnight monitoring during late pregnancy poses practical and ethical challenges, including physical discomfort, limited access to sleep laboratories, and the risk of sleep disruption. In contrast, HSAT offers a more feasible and less invasive approach with higher maternal acceptability, and its use is supported in pregnancy research when signal quality is adequate [43,44].

Following established clinical thresholds, OSA was defined as an AHI ≥ 5 events/h. The HSAT parameters included AHI, minimum oxygen saturation, and the oxygen desaturation index (ODI) [45]. All analyses were conducted according to the manufacturer’s standard procedures.

By using HSAT instead of PSG, this study prioritized participant comfort and recruitment feasibility, aligning with the exploratory and ethically sensitive nature of maternal research.

### 2.4. Assessment of Physical and Dental Development of Offspring at 3 Years of Age

At 3 years of age, children’s physical growth was evaluated by measuring their height, weight, head circumference, and one-leg standing time with eyes open [46,47]. Assessments were performed by two trained examiners using standardized methods. Oral examinations were conducted during the 3-year-old health checkup by a licensed dentist, following the guidelines of the Japanese Ministry of Health, Labour, and Welfare. The presence of malocclusion (e.g., crossbite or open bite) was recorded [34].

### 2.5. Assessment of Offspring Psychological Development at 3 Years of Age

A licensed clinical psychologist blinded to maternal OSA status administered the New K-Type Developmental Test [48]. This structured test provides developmental indices across four domains: posture/motor, cognition/adaptation, language/social, and the total composite index. Each domain includes multiple age-appropriate behavioral tasks (e.g., fine motor coordination, problem-solving, and language comprehension) that yield raw scores, which are subsequently converted to standardized developmental quotients (DQ; mean = 100 and SD = 15) based on age-specific norms, where higher scores indicate more advanced development. The total developmental index represents the average of the four domain scores.

Evaluations were conducted when the children were between 3 years 0 months and 3 years 3 months old to minimize interindividual variability [49].

The use of an examiner-administered test ensured greater objectivity and reliability compared with parent-reported questionnaires.

### 2.6. Statistical Analysis

Statistical analyses were conducted using SPSS version 30.0.0.0 (IBM Corp., Armonk, NY, USA). Data are summarized as means ± standard deviations (SDs). All analyses were descriptive and exploratory in nature, reflecting the pilot design and small sample size. Two-tailed tests were used, with *p* < 0.05 indicating a trend and *p* < 0.01 considered statistically notable.

Participants were categorized into the OSA and non-OSA groups for group comparisons. Mean (SD) values for children’s physical growth indices were compared, and malocclusion prevalence was assessed using the chi-squared test. Correlation analyses explored associations between maternal AHI and developmental indices, both including and excluding one extreme outlier (maternal AHI = 69.3).

Given the limited number of OSA cases, no formal multivariable adjustments were performed. Instead, sensitivity analyses excluding the outlier were conducted to evaluate robustness. The findings were interpreted as exploratory trends rather than confirmatory evidence. A conservative threshold (*p* < 0.01) was applied to minimize false-positive interpretations.

## 3. Results

### 3.1. Study Population

**Figure 1:**
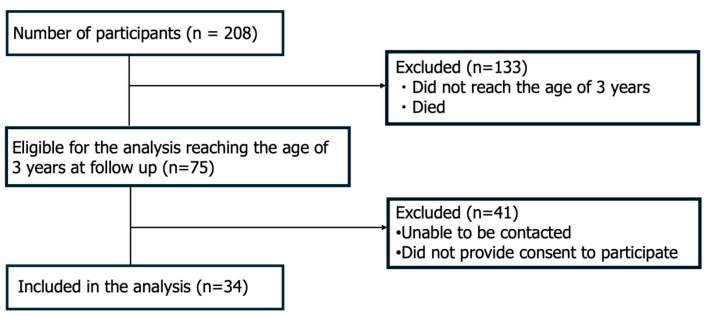
CONSORT-style flow diagram showing participant recruitment, follow-up, and analysis information.

Initially, 208 pregnant women participated in the maternal sleep study and completed home sleep apnea testing (HSAT) during pregnancy. Follow-up developmental assessments for their children were conducted when they reached 3 years of age, between 28 November 2024, and 18 July 2025. Eligible children were those born between 27 July 2021, and 15 July 2022, because only those who had reached 3 years of age by July 2025 could be assessed. Of the 208 mothers, 75 had children who reached the 3-year follow-up point within this period. Among these, 41 were excluded because the mothers could not be contacted or declined to participate in the developmental evaluation. Consequently, 34 mother–child pairs were included in the final analysis (Figure 1). Maternal sleep-disordered breathing was assessed based on the apnea–hypopnea index (AHI) measured at 28–32 weeks of gestation using HSAT, with a mean AHI of 4.4 (range, 0.0–69.3). Among the six women diagnosed with OSA, five were classified as having mild OSA (AHI 5–14.9), none had moderate OSA (AHI 15–29.9), and one had severe OSA (AHI ≥ 30).

Six women (17.6%) met the diagnostic threshold for OSA (AHI ≥ 5). Ongoing follow-up of the remaining cohort continues as part of the broader longitudinal study, but the present analysis was restricted to participants who had completed both maternal and child assessments within the defined timeframe.

### 3.2. Physical Growth Outcomes

The mean anthropometric measurements at 3 years of age were as follows: height, 93.5 cm; weight, 14.0 kg; and head circumference, 49.3 cm. Almost all children (94.1%) had measurements within the reference standards for Japanese 3-year-olds. Deviations in height, weight, and head circumference were observed in two cases each. Notably, the child of the mother with a very high AHI (>60) had anthropometric values within the normal range.

Anthropometric measurements were interpreted using the updated Japanese growth reference charts developed by Kato et al. (2014), which provide sex-specific means, standard deviations, and LMS parameters that allow age-adjusted z-score calculation [50]. These charts are population-based growth references derived from nationally collected observational data and therefore differ from prescriptive growth standards, such as those established from idealized or international cohorts. Although z-scores were not used in the present statistical analysis, this decision was due to methodological reasons: (i) anthropometric outcomes were not the primary endpoints of this study; (ii) the small sample size (n = 34), particularly the limited number of OSA cases, limits the interpretability and statistical stability of z-score-based comparisons; and (iii) nearly all measurements fell within the normative range, with only a few minor deviations. For these reasons, raw values were used to describe group differences, while the inclusion of the reference chart citation allows readers to contextualize the measurements within population-based Japanese growth norms.

No statistically significant differences were observed between the OSA (n = 6) and non-OSA (n = 28) groups (all *p* > 0.05): height, 92.3 ± 2.34 vs. 93.8 ± 4.39 cm (95% CI: −4.18 to 1.25, *p* = 0.267); weight, 13.7 ± 1.32 vs. 14.1 ± 1.65 kg (95% CI: −1.81 to 1.03, *p* = 0.552); and head circumference, 48.6 ± 0.78 vs. 49.4 ± 1.86 cm (95% CI: −1.82 to 0.16, *p* = 0.096).

### 3.3. Occlusal Status

The occlusal conditions were categorized into seven types. Normal occlusion was the most frequent for both groups (60.7% and 66.7% for the non-OSA and OSA groups, respectively). The distributions of malocclusions, including crossbite and crowding, did not differ significantly between the groups (χ^2^ test, *p* = 0.565).

### 3.4. Associations Between the Maternal AHI and Developmental Indices

Correlation analyses revealed moderate negative associations between the maternal AHI and the scores of the total developmental index (r = −0.55, 95% CI: −0.75 to −0.26, *p* < 0.01), the cognitive/adaptive domain (r = −0.59, 95% CI: −0.77 to −0.31, *p* < 0.01), and the language/social domain (r = −0.54, 95% CI: −0.74 to −0.25, *p* < 0.01). No correlation was observed for the motor/postural domain (r = −0.07, 95% CI: −0.40 to 0.28, *p* = 0.70) scores.

One case with an extreme maternal AHI of 69.3 exhibited disproportionately low developmental scores (cognitive/adaptive, 35; language/social, 31; and total, 42), despite having a normal motor/postural score (100). This outlier had a strong influence on the overall correlation trend.

**Figure 2:**
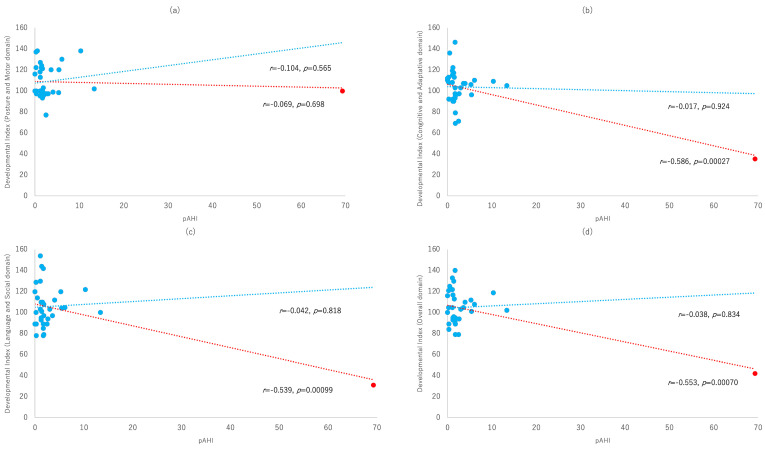
Comparison of correlations between the maternal pAHI and developmental indices with and without the extreme outlier (pAHI = 69.3). (**a**) Motor/postural domain; (**b**) cognitive/adaptive domain; (**c**) language/social domain; and (**d**) total developmental index. The red regression line represents the associations when including all participants, whereas the blue regression line represents the associations after excluding the extreme outlier (pAHI = 69.3). Pearson correlation coefficients (r) and *p*-values are shown on each graph. In analyses including all participants, the correlations are as follows: motor (r = −0.069, *p* = 0.698), cognitive/adaptive (r = −0.586, *p* = 0.00027), language/social (r = −0.539, *p* = 0.00099), and total (r = −0.553, *p* = 0.00070). After excluding the outlier, the correlations are as follows: motor (r = 0.104, *p* = 0.565), cognitive/adaptive (r = −0.017, *p* = 0.924), language/social (r = 0.042, *p* = 0.818), and total (r = 0.038, *p* = 0.834).

The descriptive distributions of the developmental indices stratified by sex, height, weight, the peripheral arterial tonometry-derived apnea–hypopnea index (pAHI), and pre-pregnancy BMI are provided in Table 1. Scatter plots with linear regression lines are provided to illustrate the associations between the maternal pAHI and developmental indices. Figure 2 presents the regression lines for all participants (red) and for the dataset excluding the extreme outlier (blue, pAHI = 69.3), allowing a visual comparison of the correlations with and without the presence of the outlier.

### 3.5. Multivariate Regression Analysis

When all 34 cases, including the extreme outlier with pAHI = 69.3, were included in the analysis, the maternal pAHI was significantly associated with the total developmental index after adjusting for sex, pre-pregnancy BMI, height, and weight at 3 years. Each 1-unit increase in the pAHI was associated with a 0.579-point decrease in the total score (95% CI: −0.899 to −0.258, *p* < 0.01). The maternal pAHI was also significantly associated with the cognitive/adaptive domain, with a value of −11.586 (95% CI: −17.91 to −5.262, *p* < 0.01), and the language/social domain, with a value of −13.163 (95% CI: −20.377 to −5.99, *p* < 0.01). As detailed in the sensitivity analysis (Section 3.6 and Supplementary Table S1), these associations were no longer statistically significant when the outlier case (pAHI = 69.3) was excluded.

No significant association was found between the motor and postural scores. The results of the univariate and multivariate regression analyses for all cases are provided in Table 2. These regression models constitute the confirmatory analyses of the primary relationships observed in the correlation analyses. Other covariates, including the children’s height, weight, and sex, were tested as exploratory factors; however, they showed no significant or consistent associations with the developmental outcomes.

### 3.6. Sensitivity Analysis

After excluding the extreme outlier (pAHI = 69.3), no statistically significant associations were observed between the maternal pAHI and the developmental indices. This indicates that the significant correlations observed in the primary analysis were substantially affected by the presence of this extreme case, rather than representing a consistent pattern across participants. Supplementary Table S1 summarizes the regression analyses without the outlier, while Supplementary Table S2 presents the developmental profile of the outlier compared with the remaining children.

In the primary analysis including all 34 participants, Figure 2 illustrates the negative correlations between the maternal pAHI and several developmental domains. However, these associations were no longer observed once the outlier was removed, highlighting the influence of this single extreme observation on the overall pattern.

In the full dataset, the developmental scores of the only case with a markedly high pAHI were lower than those of most other participants. While this observation contributed to the statistical significance in the primary analysis, it should be regarded solely as a descriptive finding within this cohort, without implying a dose–response trend or threshold pattern.

Taken together, the results do not demonstrate a stable association between the maternal pAHI and developmental outcomes in this sample. These findings underscore the impact of an extreme data point and highlight the need for studies with larger numbers of moderate-to-severe OSA cases to determine whether similar patterns appear in broader populations.

## 4. Discussion

This prospective cohort study suggests preliminary, hypothesis-generating evidence that maternal OSA during pregnancy may be associated with lower developmental scores, particularly in the cognitive/adaptive and language/social domains, in an Asian population.

No significant differences in physical growth or occlusal status were observed. However, the maternal pAHI showed a consistent trend toward association with lower higher-order developmental outcomes. These results build on previous research—which primarily centered on perinatal complications such as preterm birth and fetal growth restriction [41,51,52]—by highlighting long-term neurodevelopmental trajectories. Importantly, our study highlights the need to move beyond short-term obstetric outcomes and incorporate neurodevelopmental endpoints into the evaluation of maternal OSA, an approach that has rarely been applied in previous studies [5,26]. As a small-scale pilot investigation, this study provides initial hypothesis-generating evidence linking maternal OSA to offspring neurodevelopment, rather than offering confirmatory conclusions.

Only a few longitudinal studies have investigated the developmental effects of maternal OSA. A Thai cohort study reported an increased risk of developmental delay in children aged up to 3 years based on parent-completed questionnaires, such as the Ages and Stages Questionnaire (ASQ) [26]. However, no Asian studies have directly examined domain-specific developmental indices in 3-year-old children using examiner-administered assessments. Our results provide important exploratory evidence and highlight the need to consider maternal SDB as a perinatal determinant of child neurodevelopment. Given the craniofacial and airway anatomy characteristics specific to Asian populations, our data may provide region-specific insights that cannot be directly extrapolated from Western cohorts. Together, these findings extend prior epidemiological observations by incorporating objective, examiner-based developmental testing, thereby enhancing both the methodological rigor and regional relevance of this exploratory study.

The observed associations align with known pathophysiological mechanisms: intermittent hypoxia, sympathetic nervous system activation, inflammatory cytokine elevation, and impaired placental function may compromise fetal oxygen and nutrient supply, thereby affecting brain development [53,54,55]. Fetal hypoxia increases the fetus’s vulnerability to hypoxic–ischemic injury after birth [56] and impairs neurogenesis, synaptogenesis, and neural network maturation [57]. The most pronounced associations were observed in the cognitive and language domains, supporting the hypothesis that OSA preferentially affects higher-order brain functions. In our study, physical growth outcomes at 3 years of age were largely preserved, whereas the cognitive/adaptive and language/social domains showed selective vulnerability to the impacts of maternal OSA. This pattern may suggest that maternal OSA is linked to specific alterations in oxygen delivery and metabolic signaling that may influence neural development, rather than causing generalized impairments in fetal nutrition. Such a mechanism is biologically plausible, as placental dysfunction has been reported to compromise trophoblast function and vascular remodeling, thereby altering oxygen and nutrient transfer without necessarily reducing overall fetal growth [58,59]. Although our study did not directly assess the placental function, the observed domain-specific developmental impairments align with the hypothesis that maternal OSA exerts its effects through qualitative changes in placental oxygen and nutrient transport. Future studies may benefit from recent advances in placental imaging and biomarker research, which could provide important tools for testing this hypothesis more directly [60].

Additionally, our findings hint at possible sex-related patterns, in which boys appeared more vulnerable to OSA-related developmental impairments than girls. This is consistent with previous human and animal studies showing that male offspring are more susceptible to hypoxia-related neurodevelopmental disruptions [61,62,63,64,65,66,67]. However, given the small sample size and limited number of OSA cases in this study (n = 6), this pattern should be interpreted cautiously and not as conclusive evidence of sex-specific effects. In addition, the influence of a single extreme case (pAHI = 69.3) as a contributing factor to this apparent sex-related trend cannot be completely ruled out. Larger, sex-stratified studies are warranted in the future.

Excluding the extreme outlier (pAHI = 69.3) substantially attenuated the observed associations, suggesting a potential threshold rather than a linear dose–response relationship. Nevertheless, this interpretation remains exploratory, as the apparent threshold effect was largely driven by a single case and lost statistical significance when excluded. While consistent with a previous study reporting cognitive effects at higher AHI thresholds (≥15) [68], it was further confirmed that larger samples are needed. The pAHI was negatively correlated with gestational age, even after excluding planned cesarean sections, consistent with meta-analytic evidence linking OSA to preterm birth [69]. This observation suggests that OSA severity, rather than its presence alone, could have clinically relevant implications for fetal development.

Maternal obesity was positively correlated with the pAHI, but it was not independently associated with developmental outcomes after multivariate adjustment. This suggests that hypoxic stress from OSA, rather than obesity, may be the primary driver of the neurodevelopmental effects. These findings are consistent with those of studies reporting increased risks of attention-deficit/hyperactivity disorder and autism spectrum disorder in the offspring of mothers with obesity. However, they highlight the mediating role of the apnea severity in these relationships [70]. Identifying pregnant women with a high BMI as a high-risk group for OSA may improve the screening efficiency. The integration of maternal BMI with emerging OSA screening tools, such as home sleep testing devices and artificial intelligence-based signal analysis, may enhance the identification of women who are most likely to benefit from targeted monitoring and intervention.

From a clinical and public health perspective, incorporating OSA screening into routine prenatal checkups may represent an important opportunity to promote maternal and child well-being. Simple screening questionnaires and portable devices, such as the WatchPAT, offer feasible options for large-scale implementation, even in community or resource-limited settings. Although their diagnostic precision is lower than that of polysomnography, their use may enable the earlier identification of at-risk women, facilitating timely referral and management. This tiered approach—broad screening followed by selective confirmation—could represent a feasible and cost-effective strategy within maternal and child health programs, especially considering the lifelong educational and healthcare implications of developmental disorders.

Our findings also emphasize the importance of considering ethnic and regional differences. The craniofacial morphology common in Asian populations may predispose women to OSA, even at lower BMI thresholds compared with Western populations [71,72]. Similarly, sociocultural practices and healthcare access patterns may shape the prevalence and consequences of OSA [73,74]. Therefore, cross-population comparisons are necessary to refine risk stratification and ensure the generalizability of the findings. By situating our data within this international context, the present study provides a valuable step toward closing this global evidence gap.

In terms of mechanisms, in addition to placental dysfunction, maternal systemic inflammation and epigenetic modifications may represent additional pathways linking OSA to offspring neurodevelopment. Intrauterine exposure to intermittent hypoxia has been shown to induce DNA methylation changes and histone modifications in fetal tissues, potentially leading to the long-term dysregulation of neural and endocrine pathways. For example, Hartley et al. (2013) reported that even short hypoxic exposure in hippocampal neuronal cultures induces lasting genome-wide DNA methylation changes, affecting genes that are critical for neural development [75]. Similarly, Ma et al. (2014) summarized evidence that prenatal hypoxia alters DNA methylation and histone modifications, leading to long-term changes in synaptic plasticity and behavior in animal models [17]. Moreover, Kim et al. (2022) highlighted that hypoxia modulates histone-modifying enzymes, thereby influencing the regulation of gene expression across mammalian systems [76]. Integrating genomics, epigenomics, and metabolomics into future research could help clarify these mechanisms and identify biomarkers that are predictive of developmental outcomes, paving the way for precision medicine approaches to maternal–fetal health.

Large-scale multi-center collaborations are essential to validate these findings and move toward causal inference. International consortia with standardized protocols could provide sufficient power to examine domain-specific outcomes and sex differences while also enabling subgroup analyses across ethnicities. In addition, randomized controlled trials testing interventions such as continuous positive airway pressure during pregnancy could determine whether treatment modifies offspring outcomes, thereby addressing the critical question of causality. Such studies would represent a logical next step in translating observational findings into clinical and policy recommendations.

The strengths of this study include its prospective design, standardized assessments of multiple developmental domains, and use of a validated portable device during pregnancy.

The study limitations include the small sample size (n = 34), particularly the inclusion of only six participants diagnosed with OSA. This limited number of OSA cases substantially constrained the ability to perform stratified analyses according to severity (mild, moderate, and severe) and reduced the precision of effect estimates. Furthermore, given the small number of events, it is possible that SDB was underestimated or misclassified, particularly for borderline or mild cases, which may have biased the associations toward the null. All participants were recruited from a single institution, which may limit the generalizability of the findings to broader or more diverse populations. The reliance on the WatchPAT device rather than polysomnography may have further contributed to misclassification, as HSAT devices are less capable of detecting arousal-related or stage-specific respiratory events compared with PSG. In addition, because only six OSA cases were available and one extreme outlier (pAHI = 69.3) had a disproportionate influence, the present findings should be interpreted as preliminary and exploratory, and the true effect size of maternal OSA may have been underestimated or distorted due to this limited sample.

Residual confounding due to the maternal background, delivery complications, and unmeasured postnatal environmental factors—such as socioeconomic status, parental education, and home environment quality—cannot be ruled out. Additionally, other important maternal or postnatal variables, including maternal mental health, dietary patterns, and breastfeeding practices, were not systematically assessed and may have contributed to the observed associations. Moreover, certain maternal demographic and perinatal factors—such as the maternal age, parity, pre-pregnancy BMI, delivery mode, and obstetric complications, including preeclampsia or gestational diabetes—were not comprehensively adjusted for and could also act as potential confounders. Furthermore, the follow-up period was limited to 3 years, and the small sample size (n = 34), with only six OSA cases, all recruited from a single institution, constrains the statistical power and generalizability of the findings. The abovementioned factors are known to influence cognitive and language development during early childhood and may have contributed to the observed associations [77]. Therefore, the results should be interpreted as exploratory and hypothesis-generating.

While these findings are hypothesis-generating, routine screening using questionnaires and portable monitoring devices during prenatal care may facilitate the early identification of high-risk cases. Collaboration with perinatal and sleep medicine specialists and intervention considerations, such as continuous positive airway pressure, may help protect the long-term developmental outcomes of children. Future research should focus on large, multi-center prospective studies to enhance the generalizability and statistical power. In particular, subgroup analyses stratified by OSA severity (mild, moderate, and severe) are needed to clarify potential threshold effects and dose–response relationships. Furthermore, future multi-center investigations should incorporate full PSG to validate these preliminary findings and to improve the diagnostic accuracy of home-based testing tools such as the WatchPAT. Such studies will be essential to confirm or refute these preliminary associations and to clarify how maternal OSA severity may relate to offspring neurodevelopmental outcomes. The incorporation of objective biomarkers—such as placental imaging, neonatal or maternal neuroimaging, and epigenetic profiling—will be essential to elucidate mechanistic pathways linking maternal OSA to offspring neurodevelopment. These approaches, combined with advances in mobile health technologies and interdisciplinary prenatal care models, represent promising avenues for enhancing early identification and support at-risk mothers and children.

Ultimately, raising awareness of maternal sleep health may serve as a feasible and impactful component of early childhood development and public health prevention strategies.

## 5. Conclusions

This exploratory prospective cohort study suggests that OSA during pregnancy may be associated with lower neurodevelopment scores among 3-year-old Asian children, particularly in the cognitive and language domains, while no significant associations were found with children’s physical growth or occlusal status. The maternal pAHI was consistently negatively correlated with higher-order developmental outcomes, and the loss of significance after excluding one extreme case (pAHI = 69.3) suggests that developmental differences may emerge only beyond a certain severity threshold, rather than following a linear pattern.

These findings suggest the potential importance of screening for SDB during pregnancy, although further validation in larger populations is required before clinical implementation. Simple questionnaires and portable monitoring devices may provide feasible approaches for future studies to identify high-risk pregnancies and evaluate whether early detection and management can improve developmental outcomes. Given the small sample size and single-center design, the present study’s results should be regarded as exploratory and hypothesis-generating, warranting confirmation in large, multi-center, and cross-cultural studies. Increasing awareness of maternal sleep health may ultimately contribute to early childhood development and preventive public health strategies.

## Figures and Tables

**Table 1:** Distributions of the developmental indices.

		Developmental Index [Average (SD)]			
		Overall	Posture/Motor	Cognitive/Adaptive	Language/Social
Sex	Boys	99.3 (21.9)	105.6 (14.8)	100.3 (24.3)	97.5 (25.3)
	Girls	106.4 (14.5)	111.2 (15.8)	103.1 (14.1)	109.2 (18.7)
Height (cm)	<93.4	105.9 (21.2)	111.1 (14.8)	101.1 (21.3)	109.5 (27.1)
	≧93.4	99.8 (15.7)	105.8 (15.9)	102.3 (18.4)	97.2 (15.7)
Weight (kg)	<14.1	103.8 (21.0)	111.7 (15.2)	102.2 (22.8)	104.9 (25.0)
	≧14.1	101.9 (16.6)	105.1 (15.1)	101.1 (16.6)	101.8 (20.7)
pAHI (events/h)	<5	104.0 (16.5)	107.1 (14.9)	103.4 (17.2)	104.7 (20.2)
	≧5	97.3 (27.9)	114.7 (17.1)	93.5 (29.1)	97.0 (33.6)
BMI (kg/m^2^)	<21.6	106.6 (16.3)	111.3 (15.0)	105.6 (14.0)	107.4 (22.4)
	≧21.6	99.5 (20.4)	105.8 (15.6)	98.2 (23.4)	99.7 (22.9)

The mean pAHI (±SD) was determined for the subgroups based on sex (male and female), height (below and above the median value of 93.4 cm), weight (below and above the median value of 14.1 kg), the pAHI (<5 vs. ≥5), and pre-pregnancy BMI (below and above the median value of 21.6). BMI, body mass index; pAHI, peripheral arterial tonometry-derived apnea–hypopnea index.

**Table 2:** Univariate and multivariate regression analyses of the total and domain-specific developmental indices. The independent variables included sex, height at 3 years of age, weight at 3 years of age, the pAHI, and pre-pregnancy BMI. Statistical significance was set at *p* < 0.01.

**Overall**	**Univariable Model**				**Multivariable Model**			
	**B**	**95% CI**		***p*-Value**	**B**	**95% CI**		***p*-Value**
Sex	0.194	−0.159	0.547	0.272	−0.62	−0.393	0.269	0.704
Height (cm)	−0.162	−0.517	0.193	0.36	−0.34	−0.701	0.021	0.064
Weight (kg)	−0.047	−0.407	0.312	0.79	0.002	−0.343	0.34	0.991
pAHI (events/h)	−0.553	−0.853	−0.253	<0.001	−0.579	−0.899	−0.258	<0.001
BMI (kg/m^2^)	−0.329	−0.669	0.011	0.058	−0.204	−0.523	0.116	0.202
**Posture/Motor**	**Univariable Model**				**Multivariable Model**			
	**B**	**95% CI**		***p*** **-value**	**B**	**95% CI**		***p*** **-value**
Sex	2.866	−2.556	8.288	0.29	1.338	−5.105	7.781	0.674
Height (cm)	−3.676	−9.035	1.683	0.172	−3.622	−10.65	3.407	0.3
Weight (kg)	−1.938	−7.413	3.538	0.476	−0.1	−6.743	6.542	0.976
pAHI (events/h)	−1.06	−6.566	4.447	0.698	−1.556	−7.796	4.684	0.613
BMI (kg/m^2^)	−0.805	−6.317	4.707	0.768	−0.432	−6.646	5.782	0.888
**Cognitive/Adaptative**	**Univariable Model**				**Multivariable Model**			
	**B**	**95% CI**		***p*** **-value**	**B**	**95% CI**		***p*** **-value**
Sex	1.403	−5.646	8.452	0.688	−3.241	−9.771	3.288	0.318
Height (cm)	−0.031	−7.098	7.036	0.993	−3.513	−10.636	3.611	0.321
Weight (kg)	−0.344	−7.41	6.722	0.922	−1.633	−8.365	5.099	0.623
pAHI (events/h)	−11.5	−17.227	−5.773	<0.001	−11.586	−17.91	−5.262	<0.001
BMI (kg/m^2^)	−7.074	−13.666	−0.482	0.036	−4.854	−11.152	1.443	0.126
**Language/Social**	**Univariable Model**				**Multivariable Model**			
	**B**	**95% CI**		***p*** **-value**	**B**	**95% CI**		***p*** **-value**
Sex	5.911	−1.962	13.784	0.136	0.225	−7.182	7.632	0.951
Height (cm)	−5.225	−13.161	2.711	0.189	−8.904	−16.984	−0.824	0.032
Weight (kg)	−1.574	−9.71	6.562	0.696	0.584	−7.052	8.22	0.877
pAHI (events/h)	−12.219	−19.087	−5.352	<0.001	−13.163	−20.337	−5.99	<0.001
BMI (kg/m^2^)	−6.368	−14.195	1.459	0.107	−3.179	−10.322	3.965	0.37

BMI, body mass index; pAHI, peripheral arterial tonometry-derived apnea–hypopnea index.

## Data Availability

The datasets used and/or analyzed in this study are available from the corresponding authors upon reasonable request.

## Supplementary Materials

The following supporting information can be downloaded at: https://www.mdpi.com/article/10.3390/children12121610/s1, Table S1: Univariate and multivariate regression analyses for total and domain-specific developmental indices after exclusion of the outlier case (pAHI = 69.3); Table S2: Mean (±SD) developmental indices with the total and domain-specific scores for the outlier case (pAHI = 69.3) and all other cases.

## Abbreviations

The following abbreviations are used in this manuscript:

AHI	Apnea–hypopnea index
BMI	Body mass index
CI	Confidence interval
HSAT	Home sleep apnea testing
OSA	Obstructive sleep apnea
SD	Standard deviation
