# Supplementary material for: Preliminary Evidence Linking Maternal Sleep-Disordered Breathing During Pregnancy to Early Childhood Development: A 3-Year Pilot Cohort Study in Japan
Source: Children (Basel). 2025 Nov 26;12(12):1610. doi: 10.3390/children12121610 (PMC12731920; doi:10.3390/children12121610)
Supplement: Supplementary file 1 [file children-12-01610-s001.zip › children-3982554-supplementary.pdf]

Supplementary Table S1. *Univariate and multivariate regression analyses for total and domain-specific developmental indices after exclusion of the outlier case (pAHI = 69.3). The independent variables included sex, height at 3 years of age, weight at 3 years of age, pAHI, and pre-pregnancy BMI. Statistical significance was set at p < 0.01.*

Overall	Univariable Model				Multivariable Model			
	B	95% CI		p-value	B	95% CI		p-value
Sex	0.116	-0.248	0.48	0.519	-0.114	-0.507	0.278	0.555
Height (cm)	-0.373	-0.713	-0.034	0.032	-0.397	-0.816	0.023	0.063
Weight (kg)	-0.198	-0.557	0.161	0.27	-0.073	-0.482	0.337	0.719
pAHI (events/h)	0.038	-0.328	0.404	0.833	0.108	-0.259	0.475	0.55
BMI (kg/m²)	-0.221	-0.578	0.136	0.216	-0.302	-0.677	0.074	0.111

Posture/Motor	Univariable Model				Multivariable Model			
	B	95% CI		p-value	B	95% CI		p-value
Sex	0.174	-0.187	0.534	0.334	0.066	-0.357	0.489	0.75
Height (cm)	-0.271	-0.624	0.081	0.127	-0.227	-0.679	0.226	0.313
Weight (kg)	-0.149	-0.511	0.214	0.41	-0.042	-0.484	0.4	0.846
pAHI (events/h)	0.104	-0.26	0.468	0.565	0.117	-0.278	0.513	0.548
BMI (kg/m²)	-0.028	-0.394	0.339	0.879	-0.058	-0.463	0.346	0.769

Cognitive/Adaptative	Univariable Model				Multivariable Model			
	B	95% CI		p-value	B	95% CI		p-value
Sex	-0.042	-0.408	0.324	0.817	-0.24	-0.646	0.165	0.234
Height (cm)	-0.182	-0.542	0.178	0.311	-0.21	-0.643	0.224	0.33
Weight (kg)	-0.169	-0.53	0.192	0.346	-0.168	-0.591	0.255	0.423
pAHI (events/h)	-0.017	-0.384	0.349	0.924	0.084	-0.295	0.463	0.653
BMI (kg/m²)	-0.259	-0.613	0.095	0.146	-0.356	-0.743	0.032	0.071

Language/Social	Univariable Model				Multivariable Model			
	B	95% CI		p-value	B	95% CI		p-value
Sex	0.2	-0.159	0.559	0.264	-0.024	-0.41	0.361	0.899
Height (cm)	-0.451	-0.778	-0.124	0.008	-0.455	-0.867	-0.043	0.032
Weight (kg)	-0.22	-0.577	0.138	0.219	-0.034	-0.436	0.369	0.865
pAHI (events/h)	0.042	-0.324	0.408	0.818	0.085	-0.275	0.446	0.631
BMI (kg/m²)	-0.163	-0.525	0.198	0.364	-0.22	-0.589	0.149	0.232

BMI, body mass index; pAHI, peripheral arterial tonometry-derived apnea-hypopnea index

Supplementary Table S2. Mean (± SD) developmental indices with the total and domain-specific scores for the outlier case (pAHI = 69.3) and all other cases.

		Developmental index [average (SD)]			
		Overall	Posture/Motor	Cognitive/Adaptive	Language/Social
pAHI (events/h)	69.3 >	104.7 (15.4)	108.7 (15.5)	103.7 (15.9)	105.6 (19.0)
	69.3	42 (0)	100 (0)	35 (0)	31 (0)

pAHI, peripheral arterial tonometry-derived apnea-hypopnea index
